# Netting the Stress Responses in Fish

**DOI:** 10.3389/fendo.2019.00062

**Published:** 2019-02-12

**Authors:** Joan Carles Balasch, Lluís Tort

**Affiliations:** Department of Cell Biology, Physiology and Immunology, Universitat Autònoma de Barcelona, Barcelona, Spain

**Keywords:** stress, stressotope, fish, teleost, plasticity, transcriptomics, phenomics

## Abstract

In the last decade, the concept of animal stress has been stressed thin to accommodate the effects of short-term changes in cell and tissue physiology, major behavioral syndromes in individuals and ecological disturbances in populations. Seyle's definition of stress as “the nonspecific (common) result of any demand upon the body” now encompasses homeostasis in a broader sense, including all the hierarchical levels in a networked biological system. The heterogeneity of stress responses thus varies within individuals, and stressors become multimodal in terms of typology, source and effects, as well as the responses that each individual elicits to cope with the disturbance. In fish, the time course of changes after stress strongly depends on several factors, including the stressful experiences in early life, the vertical transmission of stressful-prone phenotypes, the degree of individual phenotypic plasticity, the robustness and variety of the epigenetic network related to environmentally induced changes, and the intrinsic behavioral responses (individuality/personality) of each individual. The hierarchical heterogeneity of stress responses demands a code that may decrypt and simplify the analysis of both proximate and evolutionary causes of a particular stress phenotype. We propose an analytical framework, the *stressotope*, defined as an adaptive scenario dominated by common environmental selective pressures that elicit common multilevel acute stress-induced responses and produce a measurable allostatic load in the organism. The stressotope may constitute a blueprint of embedded interactions between stress-related variations in cell states, molecular mediators and systemic networks, a map of circuits that reflect the inherited and acquired stress responses in an ever-changing, microorganismal-loaded medium. Several features of the proposed model are discussed as a starting point to pin down the maximum common stress responses across immune-neuroendocrine relevant physiological levels and scenarios, including the characterization of behavioral responses, in fish.

## Introduction

When studying the adaptive ecophysiology of stress in teleosts, the largest group of fishes and therefore of vertebrates, their extremely diverse life stories appear. This diversity impedes a unified and common description of stress-related effects of environmental insults in fish, and, in consequence, is understandably overlooked in comparative interspecies analyses of stress physiology. Often, the physiological effects of stressors are treated as species-specific features of the chosen animal, but not always expressly acknowledged as such. Therefore, in the literature, the uncovered stress-related feature of a single or few species becomes, misleadingly, a prominent characteristic of *all* teleosts.

**Figure 1:**
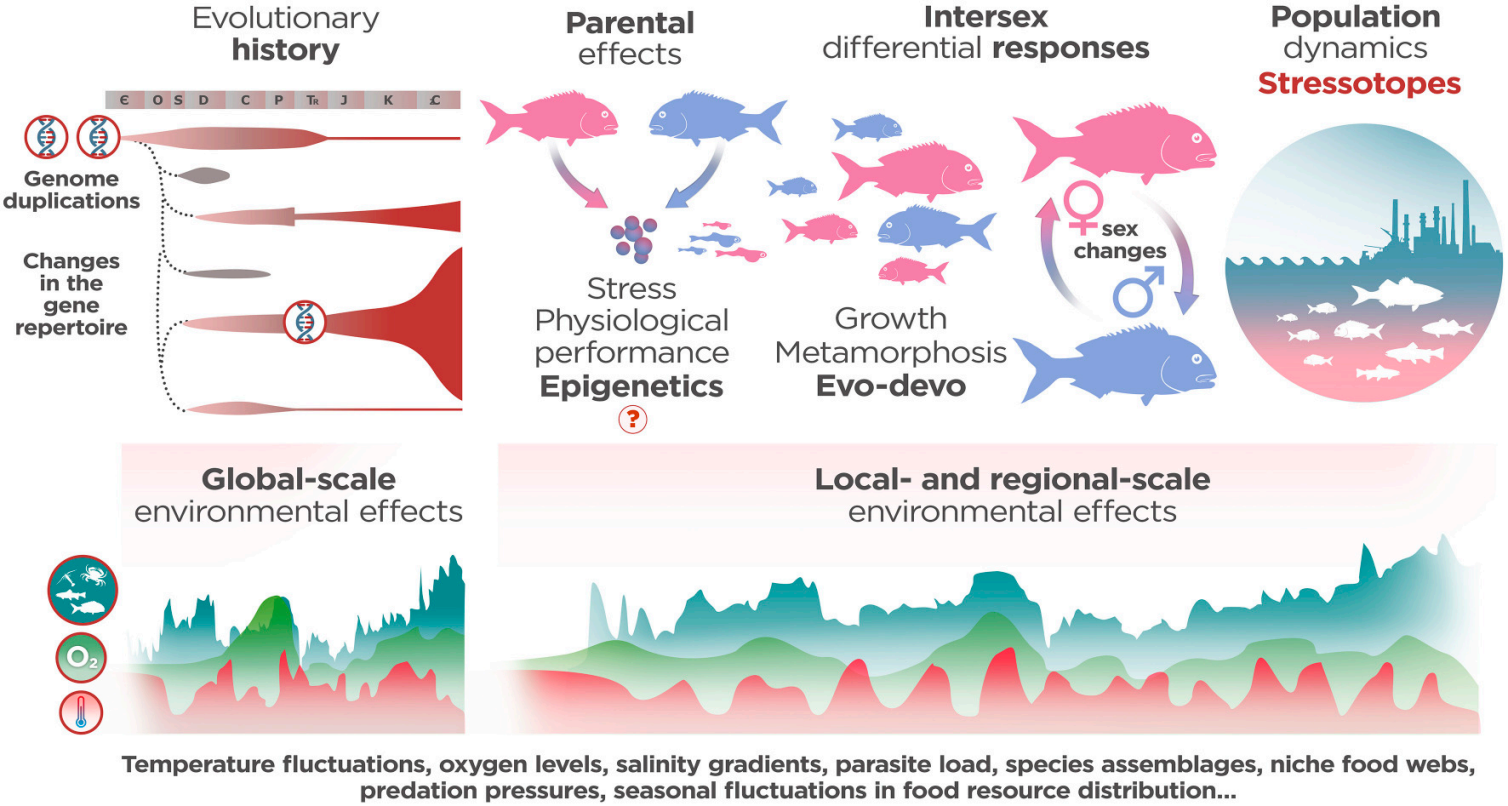
Several influences that shape distress-related phenotypes in fish. The analysis of common responses to stress relies on (1) the evolutionary life-stories endured by each species (i.e., genome duplications, changes in stress-related gene pools, changes in oxygen and temperature levels over geologic time and dynamics of extinction/speciation throughout Earth's history) that constraint the evolvability of biological systems and (2) the pattern and scale of environmental effects in a particular biotope. When analyzing a particular stress-related phenotype the physiological outcomes of specific gene networks during the twin processes of development and growth/metamorphosis have been taken into account, but also the effects of epigenetic transmission of cortisol sensitivity, the differential responses to stressors between sexes and the behavioral interactions within populations as starting points. In this sense, a stressotope defines the boundaries of common pan-specific maladaptive stress responses in a particular/local biotope not only from the perspective of abnormal changes in environmental resources, but also, from the global-scale changes recapitulated in the life story of each individual, i.e., the functional genomics and phenomics of stress intertwined with functional ecology across spatial scales.

Reducing the exogenous and endogenous covariates that elicit stress-related responses undoubtedly helps to reproduce a more focused physiological process in the laboratory. However, this approach veils the adaptive and, more importantly, *content-rich* interactions between stress-related gene expression and phenotype turnover across the life stories of each species. Consequently, the high diversity of teleost lifestyles enriches the physiological analysis of stress effects in fish, but also flaws a unified description of common responses to stress. To overcome this dilemma, the analysis of pan-specific common predictors of stress-related responses should be entrusted to the accurate selection of more explanatory variables. For example, when analyzing the effects of high or low temperatures on physiological performance in ectothermic species, choosing species-specific optimal temperature limits (thermopreferundum) as baseline values allows for comparing the effects of common stressors (1, 2). This approach assumes that the thermic reference summarizes the adaptive pathway to temperature tolerance evolved in a particular biotope (and, implicitly, part of the adaptive life story of each species), and guarantees a more realistic description of the “natural” (or *eustressed*, see below) vs. maladaptive (*distressed*) pathways of stress responses. The same applies for the comparative inter-species analysis of immune responses to stressors in adult fish, where we should consider specifically the maturation of primary and secondary immune organs rather than the relative size of fishes. The microorganism load may substantially differ between marine and freshwater realms, but both environments share the deleterious effects of the communities of resilient low-abundance pathogens (3). Therefore, diverse stress-related physiological adaptations in teleost inhabiting aquatic biocenosis are to be expected, as well as the inter-species commonalities of biological signal transduction and physiological axes. Given that, the degree of functional maturation of immune-related organs and tissues becomes a proxy for adult/mature physiology and allows for the effective cross-species comparison of immune responses to stress in a microbial-rich environment. These examples suggest that when we analyze a particular stress-related phenotype we are not only describing the physiological outcome of specific gene networks, but also the recapitulation of the evolutionary life-stories of each individual (Figure 1).

Considering the complex influences between environmental stressors and pathogen communities, in this short review we propose a modified biotope concept (4) for analyzing stress-induced *abnormal* responses (i.e., capable of inducing an allostatic load that compromise the evolutionary conserved activation of regulatory stress-related physiological axis responsive to normal/adaptive stress, see below). This approach would reduce the complexity of species-specific stress analysis to a set of common descriptors, endogenous and exogenous, of such responses. Here, we define a teleost “stressotope” as an adaptive scenario dominated by common environmental selective pressures that elicit common multilevel severe stress-induced responses and produce a measurable allostatic load in the organism.

Defining the components and dynamics of a stressotope may help to reframe the variability of interspecific stress responses in teleosts in terms of the cross-linked interactions between niche characteristics, diverse genomic scaffoldings and phenotypic specificities that define a set of common, multilevel stress responses in fish. Several endogenous and exogenous features that may be relevant to modeling stressotopes are presented below as a starting point, by no means exhaustive, to discuss the value of this ecophysiological approach to analyze the commonalities to stress responses.

## Stressing the Stress Responses

Although some definitions and general considerations on the stress concept involve the idea of an altered status and physiological exceptionality, it is also true that coping with stressors, the stress course, and the response of the organism are not only a common mechanism but also a very sound and conserved response among living species. Hence, the stress responses should be considered as one of the basic and important mechanisms that are key to maintain the physiological, cellular and molecular stability (*homeostasis*) of the organism. A myriad of mechanisms available to face the impact of stressors will be selected or modulated depending on many factors: the species itself, the environmental conditions, and chiefly, the intensity, duration and predictability of the stressor. Therefore, an important part of the machinery behind the stress response is the same that is engaged after other stimuli that are not considered stressors, such as reproductive changes, exercise, immune stimulants, feeding, light-dark transitions or the presence of conspecifics or enrichment objects. That is why it is also difficult to make a definition of the stress concept with precision.

Along the years and among the authors that have dealt with the concept of stress (5), several definitions have been provided following the initial definition, “the non-specific response of the body to any demand placed upon it” that was proposed by Hans Selye in 1951 (6). Several concepts have been proposed that agree with the current consensus that stress responses emerge when the stimulatory demand exceeds the natural regulatory capacity of an organism (7). For instance, Selye's *eustress* and *distress* (8) responses differentiate between a “normal” state, in which no significant alterations are recorded and the homeostasis is not impaired (although some hormonal, metabolic or molecular stress-related mechanisms can work), and an “abnormal” state in which significant alterations are regarded, an overall perception of alarm occurs and the stress-related mechanisms are highly engaged. *Hormesis* has been defined as any process in which a cell or an organism exhibits a biphasic response to exposure to increasing amounts of a specific condition (9). It is currently applied to chemical stimuli but it has been applied to amounts of sensory stimulus, metabolic alterations and stressors. Thus, low-dose exposures would elicit a stimulatory, beneficial or compensatory response (eustress), whereas high doses elicit inhibition, alteration or suppression (*distress*). Likewise, the term *allostasis* (10), refers to a concept linked to the energetics or the “economy management” of the body resources. Any stressor may lead to an allostatic load that first, compromises the overall balance of the organism, and second, involves a higher demand of resources that either leads to a higher acquisition of food/energy or induces a number of physiological and metabolic internal compensations in order to retain the lost balance. This results in maladaptation, which indicates that the regulatory mechanisms have not been able to compensate the effects of the stressor. Maladaptation is often associated to chronic stress since heavy acute stressors may result in death, and mild ones in recovery. These chronic stressors leading to maladaptation are very relevant in farmed animals, including fish subjected to artificial conditions.

The *perception* of stress involves the receptor-mediated sensing of the stressor, either physiologically at neuro-endocrine or cellular levels. The perception mechanisms are important, not only to act as transducers of alarm signals but also to discriminate the intensity of the stress stimuli and therefore the threshold required to trigger the response mechanisms. In fish, neuroendocrine signaling affects and becomes regulated by the onset of immune responses, due to the peculiar organization of the head kidney, a hematopoietic tissue made from a mixture of endocrine, hematopoietic and immune cell populations, akin to the mammalian adrenal gland and bone marrow. As in the rest of vertebrates, those responses are mainly mediated by the activation of two hormonal axes in fish, the sympatho-chromaffin (SC) axis and the hypothalamic-pituitary-interrenal (HPI) axis (11). The SC axis activates a fast stress response, involving the cardio-respiratory system by increasing ventilatory and heart rates, heart stroke volume, and blood perfusion in gills and muscle, providing glucose supply to critical tissues, with adrenaline being one of the major mediator hormones. An activated HPI axis contribute to the re-organization of resources by increasing the catabolic pathways, supplying glucidic sources, processing fatty acids for energy, and suppressing other high-cost energy and longer-term processes such as those of immune responses, being plasmatic cortisol levels one of the major mediators (12).

By binding to glucocorticoid (GR) or mineralocorticoid (MR) receptors, cortisol regulates neuroimmunoendocrine circuitries elicits stress-induced immunosuppression and contributes to allostatic imbalances. That is why is particularly suited for stress-related surveys in natural and artificial environments and the focus of the search for common global markers of stress states in fish. However, the levels of cortisol in distressed fish and, consequently, the individual perception and physiological effects of the intensity of the stressors, are usually strongly biased for neuroendocrine and immune systems in a highly species-specific manner, which makes the prognosis of stress recovery both apparently simple and dauntingly complex (13). Moreover, within-species diversity in cortisol levels also differs between behavioral phenotypes. As discussed below, selecting for “bold” (proactive) and “shy” (reactive) individuals in a population also segregate animals as low- or high-cortisol responders, masking the common cortisol-related responses to stress. A side effect of this behavioral phenotyping can be seen in experiments with paired trout, in which agonistic competition for food resources leads to cortisol-based hierarchical social labeling, with animals ranging from dominant (proactive, usually with low plasmatic cortisol levels) to subordinate (reactive, usually with high plasmatic cortisol levels). When the social status is reversed, cortisol levels in former subordinates are recovered quickly, rendering useless the measure of cortisol levels as a global long-term common marker of social stress (14). The direct effects of social status on plasmatic cortisol levels should also be balanced out by analyzing the food control exerted by the dominant conspecifics that may indirectly elevate cortisol levels in food deprived stressed subordinates.

Cortisol implants may fail to act as a proxy of behavioral patterns in teleosts (15, 16), and the repeatability of cortisol profiles is higher in reared as opposed to free-living fish due to the artificial control of environmental variables (17). Circadian and seasonal cycles of cortisol secretion must also be considered for assessing the sensitivity and adaptability to stressors (18), considering that cortisol are involved in the synchronization of circadian systems in fish (19). This clearly indicates that a more complex multiscale approach (i.e., from cellular activation to organism and population dynamics in specific stressotopes) will be desirable to describe the effects of stressors.

Besides cortisol, other mediators of stress responses, namely major regulatory axis components (ACTH, CRH, proopiomelanocortin –POMC- peptides, β-endorphin, α-MSH), opioids and a myriad of immune cytokines have been extensively used to define commonalities in altered stress states, but the species bias remain. In the last decade the quest for commonalities of stress responses in fish has focused in peripheral structures such as the mucosae, that sense and distribute alarm signals from pathogens, parasites, bacteria, injuries, sudden changes of salinity or oxygen or the presence of chemicals in the water (20, 21). Skin, gills or intestine may often be the first structures that sense the stressors, but they do so again in a marked species-specify manner (20, 22, 23). The reorganization of the overall metabolism to cope with the stressors also involve an alteration of thyroidal axis (24) related to the energetics and mobilization of fat resources, especially in fish undergoing severe metamorphosis regulated by environmental shortages, such as in smolting salmons (25, 26). Under stress, growth is arrested, the reproductive processes are suppressed or depressed and chronic stressors induce immune suppression, in particular in expensive processes such as white cell production and antibody production, whereas other responses such as phagocytosis may be maintained (27, 28). However, as seen in whole organism physiological responses, at the cellular level the delicate equilibrium between adaptive and maladaptive stress seems to be the norm. Reactive oxygen species (ROS), for example, signal oxidative stress as an evolutionary conserved phagocyte response to infection or xenobiotics (29). However, as part of the environmental stress response, the expression of ROS-related genes vary in hermetic fashion: mild oxidative stress promote the expression of antioxidant defenses that, if defeated, lead to enhanced gene expression that may have distressed outcomes (30). The effects of stress-essential (responsive to specific stressors) and stress-induced (involved in metabolic and high order neuroendocrine axis activation) genes (31) reach from cellular disturbances all the way up to systemic processes, and demand a multilevel approach to determine stress sensing and resolution in a stressotope context.

Notwithstanding the intensity of the stressor, in fish as in other vertebrates, the onset of short-term stress mechanisms usually correlates with genome-fixed and protective adaptive responses to seasonal and predictable environmental perturbations and health insults, whereas long-term responses to stressors tend to be considered as harmful expressions of allostatic imbalances in an unpredictable or pathogen-ridden environment (32). This brings the necessity for a broad multilevel framework that may define more precisely the effect of stressors in cellular, physiological, pathological/clinical and (eco)systemic scenarios.

## Overcoming the Scenic Fear

Ancient and extant biotic and abiotic dynamics of aquatic environments shaped the adaptive/essential stress responses of fish in a species-specific fashion and should be considered when defining a stressotope. Here we discuss the effects of environmental stressors from a dual perspective, including the physical heterogeneity (natural and man-made) and the reeducation of genomic landscapes in populations placed under explicitly perceived predation risk.

The term “fishes” continue to be a phylogenetic trap that encompass a loosely grouping of more than 28,600 species of ray-finned fish (Actinopterygii) and elasmobranchs, unequally distributed in freshwater (12,740 species) and marine (15,886 species) environments (33). The distribution and diversity of life story patterns in extant fish reflect the differential characteristics of both realms that helped to shape the organization and expression of stress-related genome structures. Teleosts comprise a monophyletic group that accounts for roughly 98% of species of ray-finned fishes. Both marine and freshwater environments seem to be dominated by percomorphs and ostariophysians (34), but marine fishes show an unexplained low diversity in a realm that covers 70% of the Earth's surface (35). Several competing hypothesis have been suggested unsuccessfully to explain such differences, ranging from ecological constrictions, homogeneity-heterogeneity of water biotopes or ocean's net primary productivity and spatial heterogeneity [see (34–36) for a comprehensive review]. Freshwater fishes inhabit a 0.01% of available planetary water volume, usually more fragmented, prone to isolation and barred to dispersal of organisms than oceanic environments (37). This favors intense selective pressures that quite frequently lead to niche-specific diversification, adaptive radiations and increasing speciation, the many phenotypes of African cichlids being the most cited example of such processes (38). It has also been described a greater resilience to extinction in these freshwater low-density, high-diversity specialized fish populations compared to their marine counterparts (39), probably due to the differential exploitation of resources (detritivores seem to be more abundant in freshwater environments) and large-scale geological perturbations. In this sense, freshwater taxa seem to be more affected and selected for temperature and climatic variations (33).

Anoxia and osmotic changes affect teleost performance, but, being fish ectothermic and oxygen levels and saline content strongly dependent of temperature, thermal conditions largely define the boundaries of stressotopes. In fish, a sudden drop in temperature diminishes the production of immune cellular and molecular resources, impairs T cell-dependent immune responses and may led to cellular inactivation or anergy (40–42). High temperatures correlate with enhanced parasite transmission and resilience within hosts' bodies (43, 44), even when the onset of behavioral fever may stimulate phagocytic activation and modulate innate humoral responses (45). In fish, shifting too far away from thermopreferendum wakes up distress-induced genes and alters the responsiveness of HPI and immune axis (46), but the overall effect may be modulated by acclimation to temperature changes (42). In this sense, the plasticity of phenotypic responses to thermic-related stressors dictates the type and relevance of physiologic variables to be included in a stressotope.

From those observations it is clear that the number and distribution of fish species and, consequently, their physiological strategies to cope with stress result from, and are influenced by the different rates of speciation and extinction (i.e., net diversification) in each environment. Several model species, such as trout, zebrafish or carps inhabit freshwater niches and may endure unexpected selective pressures due to the limitations of toxic drainages, xenobiotic clearance or dissolved oxygen-consuming autotrophic blooms, common to lentic environments. Under these conditions a high turnover of species richness, together with accelerated evolution of stress-related homeostatic mechanisms is to be expected. For example, in fast-growing short-lived killifish species, the exposome, defined as an adding-up response to a lifetime expositions to environmental insults (47) correlates with a fast paced adaptation to Human Induced Rapid Environmental Changes, HIREC (48). Complexity, severity and pace of HIREC changes have been proposed to explain the rapidly acquired tolerance to stress of different populations of killifish (*Fundulus heteroclitus*) in polluted estuaries (49). In this species, a maladaptive stress scenario forced the emergence of genetic polymorphisms related to xenobiotic clearance and stress responses such as the aryl hydrocarbon receptor (*ahr*) signaling pathways, cytochrome P450 1A (*cyp1a*), heat shock proteins (*hsp70*), multidrug resistance transport proteins (*mrp*) and estrogen receptors (*esr2b*). In this model of distress modulation, the environmental trade-offs defined a pattern of gene expression and the emergence of low-responders stress-tolerant populations, but the fitness costs depended on specific particularities of newly adapted phenotypes. This suggests that the physiological costs of evolving tolerances to specific stressors strongly depend on the population and individual fitness in a particular niche. In other words, in diversified population assemblages, well-characterized and common stress phenotypes expressed from stress-related genetic markers may quickly reverse in a population-specific manner, hindering the definition of a set of common stress genes. Moreover, the expression of gene regulatory networks observed in different populations of killifish was complex enough to preclude a one-to-one relationship between clusters of expressed genes and adaptive features of observed fish phenotypes (50), probably due to the heterogeneity of xenobiotic stressors. Even so, under strong selective pressures, convergent evolution may favor the expression of a handful of stress-induced genes (51, 52), shared among populations and, possibly, species. This may be useful for the purposes of establishing a common set of pan-specific responses to different stressors in fish.

The effects of chronic stressors are context-dependent and involve a long-term activation of HPI, SC, and other physiological axis (reproductive, immunological, thyroidal/metabolic) influenced by stress. In the quest for rationalize and simplify stress responses across species, an even more applied definition of stress may help (53): *perceived* anticipatory stress, acute or not, resulting from continuous predation risk. Laundré's “Landscape of Fear” (LoF) defines this perceived stress considering the risks of foraging in unsafety habitats (54). Predation risk, parasite load, metabolic trade-offs associated to seasonal resource shortages, living in high density populations, or artificial habitats, HIREC influences and evolved life story traits have been used to frame the stress related to a particular biotope, usually measuring behavioral patterns and glucocorticoid levels as distress indicators (55, 56). However, despite the content-rich description of these analyses, few studies have approached the effects of LoF in fish. Behavioral cascades and patterns of risk aversion have been documented in coral reef fishes (57–59) and juvenile salmonids (60). In a highly simplified model of predator-prey relationship between trout (*Oncorhynchus. mykiss*) and its prey, (*Daphnia pulex*) in a salinized environment coupled with alarm kairomones, osmotic stress diminished the predatory pressure and favored prey abundance, whereas alarm cues reduced trout aggression (61). The effects of combined stressors, however, did not affect trout growth, probably due to the limitations of the model.

The individual's perception of stress may also collide with the maladaptive effects of HIREC-related ecological traps. Albeit scarcely studied in fish, man-made changes in an otherwise low-quality habitat may attract fishes unable to properly evaluate the amount of resources available. As a result, a behavioral glitch may lead to a struggle to survive in an “evolutionary trap” (62). For example, drifting fish aggregation devices act as supernormal stimuli (63) and may lure tuna species to misinterpret habitat resources (64); coho salmon (*O. kisutch*) prefer spawning habitats that greatly reduce their survival (65); and increased water acidification confounds visual cues in damselfish (*Pomacentrus amboinensis*) reducing their antipredator responses (66).

**Figure 2:**
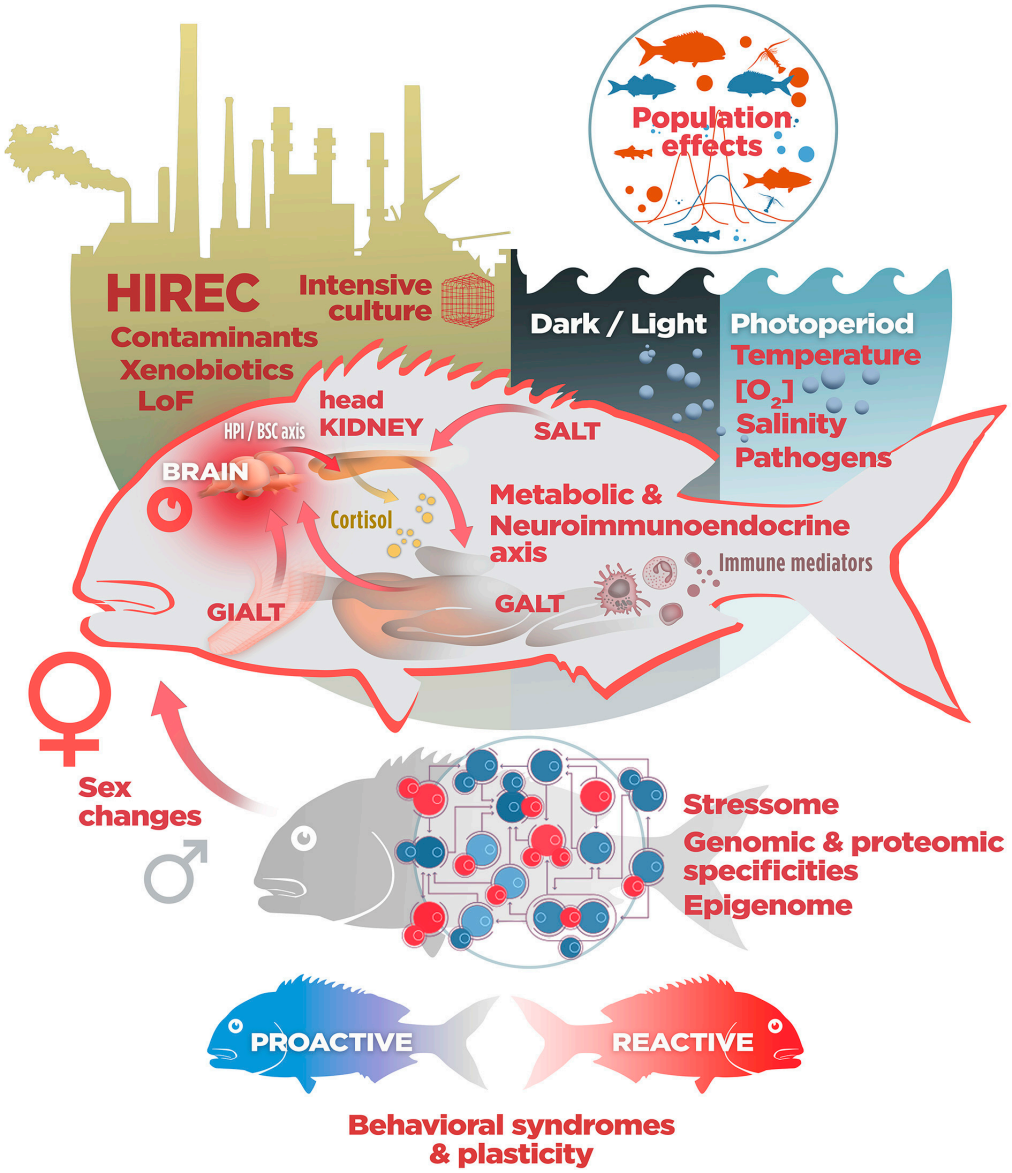
A non-exhaustive list of stressotope components. In fish, the fate of stress responses to natural (temperature and oxygen variations, changes in salinity and photoperiod, abundance of pathogens in freshwater and marine realms…), artificial (cultured) and Human Induced Rapid Environmental Changes (HIREC) depends mainly, but not only, on environmental insults, perceived stressful scenarios influenced by continuous predation risk (Landscape of Fear, LoF) and species-specific intersex differential activation of stress, immune and metabolic axes. To what extent phenotypic plasticity helps to cope with maladaptive stressors in turn depends on evolutionary conserved life stories and behavioral repertoires (see the text for details and abbreviations).

Taken together, those studies confirm not only that the complexity of the stressotope should be assessed against a minimum common number of informative variables (Figure 2), not restricted to binary food webs, but also the relevance of *eco*physiological approaches to describe a unified response to stress in teleosts. Both net diversification and the effects of perceived risk of depredation and foraging in natural and artificial habitats provide a coarse-grained description of environmentally-related impacts on stress physiology in teleosts and may help to discriminate shared mechanisms common to stress responses in fish, but the historical genomic remodeling must also be considered.

## Rolling Genomes

To delineate a stressotope, a set of pan-specific genes involved in maladaptive responses to stress must be defined. In the seascape of fish phenomes, genomes are being continuously tested and polished against the evolutionary coupling between environmental and endogenous selective pressures. This affects specifically the recent omics interpretations of adaptive physiology of stress in fish. In less than a decade, stress studies have evolved from moleculocentric analysis to genocentric approaches, and lately, to genome-wide association studies, proteomic analysis and high throughput genomic interpretations of genetic and epigenetic networks' cross-talking with environmentally-induced phenotypes that have been thoroughly reviewed elsewhere (67–70). Dissecting genome-based responses to severe stressors implies an extensive analysis of gene regulatory networks and interactions in cellular and tissue environments. To make the analysis of genome-phenome interactions more manageable, we can define a “stressome,” or catalog of genes and its products expressed when the organism suffers a maladaptive stress, a concept borrowed from studies of microbial resistance to stressful insults (71) that has been coined to characterize the roadmap to stress-related changes in genomic, proteomic, and metabolomic arenas (72, 73). Stressomes pave the way to a precise definition of stressotopes, but several methodological and conceptual issues have arisen in the course of the genocentric turn of fish stress physiology, mainly the scarcity of model species and the peculiarities of fish genomes that affect their expression, plasticity and evolvability under maladaptive scenarios.

Several species of teleosts are considered the gold standard for developmental, evo-devo, stress-related, and toxicogenomic studies (20, 74–80). However, to date <0.5% of those species have a detailed, but still far from being systematic, coverage of genomic data (81). From the vantage point of comparative studies, teleost genomes differ from those of other vertebrates in terms of divergence and redundancy. In addition to the two events of whole genome duplication common to early vertebrates, teleost endured another round of teleost-specific genome duplication 320 million years ago (Mya) (82). Some lineages widely used as model species, such as Salmonidae and Cypriniformes have experienced yet another process of tetraploidization, ~80 and 8 Mya, respectively (83, 84). To what extent this diversification leads *per se* to increased phenotypic plasticity and adaptability to environmental stressors by means of neofuncionalization of duplicated genes is still controversial (85, 86), being the subfuncionalization (the functional division of ancestral genes among the duplicated ones), loss of genes or slow evolution of duplicate genes three major outcomes of genome duplication (87, 88). For example, the recent (<10 Mya) independent evolution of anadromy in salmonid clades has been correlated to cooler temperatures that opened new estuarine and freshwater habitats, and also redefined previous stressotopes, favoring speciation (85). As described for extremely diversified non-tetraploid cichlids, several ecophysiological factors may influence a successful radiation to stressful environments without specific genome duplications. Instead, genome-wide diversifying selection on key genes, gene duplication and regulation by microRNAs and transposable elements may have allowed their adaptive radiation (89). Additionally, the teleost genomes analyzed to date seem to have suffered accelerated rates of nucleotide divergence, high rate of intron turnover and dramatic loss of conserved noncoding sequences and *cis*-regulatory elements [see (90) for a comprehensive review] that may contribute to their great phenotypic diversity in response to stressful ever-changing environments. However, this may impair the inclusion of a set of common stress-related genes as required when defining a stressotope.

This implies that the species-specificity biases the comparative genomics of teleosts, but a stressome made of a set of common predictors of distress still can be assembled from genome-wide analysis. This is the case for annual killifish genomes that contain several *hsp* transcripts and genes associated with mitochondrial function that confer resistance to severe (and more importantly, predictable) environmental anoxia stress during development and diapausa stages (91). Atlantic cod (*Gadus morhua*) also has a surprisingly high number of major histocompatibility complex (MHC) I genes that supply the absence of MHC II components, thus maintaining functional antigen trapping and processing pathways during the onset of immune responses (92, 93) in microbial-rich environments. Despite their disparate life stories, metabolism, longevity and genome scaffolding, both species can still act as genomic models and source of candidate predictors for distress-related markers because the processes evaluated (the extreme stress tolerance and the alternate antigen processing) recruit enough identical or very similar categories of predictors for an effective description of a common stressome. Gene expression profile outcomes may differ between stressors and species, and the methodology is certainly not without pitfalls [see (94, 95) for a detailed discussion], but including the adaptive life stories and the environmental biotope may normalize the analysis of physiological responses to distress. For instance, uncovering the seasonal oscillations of stress-related regulatory networks may help to define stressotopes in a more realistic way. Cortisol has been shown to induce the expression of *per1a* and *per1b* and repress *bma11a* and *clock* genes that control circadian rhythms in fish, and it has been proposed to act as a modulator of molecular oscillators (19, 96). Molecular clocks that respond to environmental factors such as light and dark cycles, food availability and thermal conditions vary both in natural and in HIREC environments and may contribute to the ticking of stressomes in a set of defined stressotopes involving migration and breeding scenarios (97).

Epigenetic modification of xenobiotic and temperature stress-related gene expression should also be considered to define a teleost stressome. Fish genomes differ from those of mammals in the number of methylated sites retained early in development and contain exclusive DNA methyltransferase genes that may help in the vertical transmission of the epigenome (98, 99), but the overall modulation of gene expression follows the vertebrate pattern (100). Epigenetic analyses have been used to test the effects of captive rearing in salmons, suggesting that hatchery-induced epigenetic changes impair the osmoregulatory seawater acclimation and swimming performance during smoltification (101). In zebrafish (*Danio rerio*), xenobiotic exposure modified methylation patterns during embryogenesis (102). Diversification of cortisol-responder phenotypes in stickleback (*Gasterosteus aculeatus*) offspring of stressed mothers has been ascribed also to epigenetic changes (103) signaled by glucocorticoid receptors. Little is known about the long-term effects of vertical transmission of stressed phenotypes in fish, but higher responses to cortisol may reduce the fitness of hatchlings and contribute to allostatic load in stressful environments (104). In addition, adaptive epigenetic modifications of gene expression strongly depend upon the degree, intensity and predictability of environmental changes that may propitiate maladaptive outcomes of epigenetic modifications, such as the epigenetic traps discussed below.

Teleost inhabit a stress-prone scenario that favors the evolution of highly reactive immunological surfaces, such as fish mucosal skin, gills, or gut, infiltrated by mucosa-associated lymphoid tissues (MALT), exquisitely sensitive to pathogenic or xenobiotic insults (21), and that's why the analysis of interfacial tissues can be so rewarding to define a stressome. Fish skin scaffolding consists of a highly secretory non-queratinized living tissue that harbors stress-sensing cells, skin associated lymphoid tissues (SALT) packed with B and T cells, resident or errand myeloid phagocytes and cells that produce microbicidal molecules and protective mucus. Teleost SALT induce and regulate local adaptive immune responses that may communicate with other mucosal tissues (branchial, GIALT, and intestinal, GALT) and influence the immune reactivity of systemic lymphoid (head kidney, spleen, thymus) and metabolic (liver) organs. In addition to immunological sensing and regulation, fish gills and gut are also involved in osmoexcretory/acid-base balance and energetic metabolism (105, 106). In fish, such multipurpose organs serve both as probes to distressful environmental changes and as effectors of allostatic rearrangements of stress-related hormonal axis, and may be specially suited to define minimum common molecular markers of distress across species. In a recent study (20), the short-term effects of hypoxia and vaccination against *Vibrio anguillarum* elicited a strongly interspecific differential response of pro-inflammatory and stress-related genes in MALT of gilthead seabream (*Sparus aurata*), a marine species, and rainbow trout (*Oncorhynchus mykiss*), a freshwater teleost, being the former more responsive to stressors. The stress- and immune-related transcripts tested (*lysozyme, c3, igm, hsp70, cox2, Il1*β, *tnf*α, *il6, il10*, and *tgf*β*1*), together with the analysis of mucosal- and plasmatic-derived cortisol levels constitute a typical set of markers of distressed states that may help to define a minimum common set of gene-driven responses to stressors in teleosts.

## Janian Phenomes

Nested in the archaic roman pantheon, a two-headed figure, Janus, represent, among other things, the transition from one state to another, or from the past to the future. In both vertebrates and invertebrates, behavioral phenotypes may change during the lifetime of an individual, following a reaction norm defined by environmental changes that enhance or suppress the expression of key behavioral mediators, and constrained by the adaptability of the genome (107). A stressotope should consequently be defined by the ontogenic variations and changing phenotypes that the organism endure in diverse environments. In teleosts, the study of relevant stressful-prone “janian” phenotypes has come to focus in recent years in the grounds of fish welfare, and include among others the ecological distribution of differentiated behavioral syndromes or individualities (“personalities”) ruled by environmental stressors [extensively reviewed in (108) and not to be discussed here], the pathogen effects on physiological modifications underlying sequential sex changes and the physiological changes linked to transition from freshwater to marine realms in diadromous species.

The majority of fish follow the usual vertebrate gonochorism, with both sexes being determined genetically or environmentally (109). Several teleosts also indulge in a plethora of rare vertebrate reproductive modes ranging from simultaneous and sequential hermaphroditism to parthenogenesis (110, 111) that have been ascribed to differential ecological selective pressures (111), diversification of reproductive mediators by means of whole genome duplication events (86) and fish-specific idiosyncrasies of gonadal axis. Males and females usually inhabit the same environment, but the selective pressures faced by both sexes may differ owing to variations in size, competition for resources, diet, microhabitat use aggressiveness and metabolic trade-offs between gamete production/fecundity and immune resistance to parasitic load (112), even in sex-role-reversed species (113).

Several sex-biased effects of parasitism and facultative infections have been described in natural and artificial populations of teleosts. Poeciliids have been used as a model to highlight the relevance of sex-specific evolution of physiological responses to environmental changes on a macroevolutioanry basis (114). Polygynous guppies (*Poecilia reticulata*) parasitized by *Gyrodactylus* spp., showed an increased responsiveness to infection in females that lead to differential evolution of resistance phenotypes (115). Male guppies also differ from females in the navigational abilities associated to increased dispersion and mobility in complex environments (116) and seems to be more prone to parasite infection than females (117). Unpredictable chronic stress (social isolation, crowding, tank changes, thermal variations, and chasing) affect zebrafish males but not females (118), highlighting the double effect of species-specificity and sex-biased covariation in stress studies. The offspring of largemouth bass females (*Micropterus salmoides*) treated with cortisol showed lower responsiveness to stress and exhibit less exploratory behavior and aggression than those of non-treated females (119), adding to the stressotope equation the still imprecisely described mechanism of vertical transmission of stress-related phenotypes.

Parasitic load and unexpected environmental changes may also contribute to the stressful effects of sex-biased physiologies. Parasite burden accounts for a large portion of stressors in aquatic habitats, and in vertebrates immunocompetence depends largely on male and female sex hormones, being testosterone generally immunosuppresive and estrogens enhancers of immune system in a broad sense (120). Vertebrate males also tend to rely more than females in Th1-mediated immune responses (linked to defensive responses against intracellular bacterial and viral parasites) whereas females display generally higher Th2-mediated extracellular responses against parasites (121). Both T-cell related immune responses have been described in fish, albeit with species-specific kinetics that may interfere or potentiate with the resistance to severe infection (122) and the intensity of distress responses. However, sex-specific responses to reproductive hormones may be altered by HIREC changes in water composition, as demonstrated by the effects of endocrine disrupting chemicals such as 17β-oestradiol in host-pathogen interaction between males and females of three-spined sticklebacks (*Gasterosteus aculeatus*) and the cestode parasite *Schistocephalus solidus* (123). When exposed to high doses of estradiol, parasitized stickleback males were found to be greatly affected, more than females by parasite growth.

A reduction of fitness in one sex has also been suggested as the trigger of selective vulnerabilities in species with environmentally-directed sex determination (ESD). Unexpected temperature changes may influence epigenetic regulation of breeding strategies in teleosts with ESD as described for mangrove killifishes (124). Similar to the “ecological traps” discussed above, severe environmental or HIREC variations could skew the sex ratio by inducing short term epigenetic changes that favor accelerated adaptation to novel environments but can become “epigenetic traps” in the long term, benefiting one sex and decreasing the fitness of the other (125). The same holds true for sequential hermaphroditic species (126), such as the extensively farmed Sparidae. Several species of this family practice protandrous (changing sex from males to females) and protogynous (the opposite) hermaphroditism (127). In protandrous gilthead sea bream (*Sparus aurata*) populations, the few large fertile females surrounded by many smaller males skew the sex ratio and have greater fitness measured by the number of offspring (128). In this species, reproductive success may be linked to the high rates of evolution of female-biased genes compared to male-biased genes (129), probably due to differential selective pressures for both sexes at each stage. This suggests that the effect of environmental stressors may affect the sex-biased expression of genes in hermaphrodites in a different way from what has been described in gonochoristic teleosts.

In diadromous species, the still poorly understood and complex influence of glucocorticoids as mediators of stress responses modulates stressome structure and function. In teleosts, crossing continental and oceanic aquatic environments stresses the physiology of osmoregulation and metabolism in a complex combination of enhancing and suppressive expression of HPI, growth and thyroidal axes. A recent study embraced the joint analysis of ontogenetic stages, sexual, and parasitic effects in hypoxia-stressed European eels (*Anguilla anguilla*), defining a limited stressotope to modulate the causes and consequences of the stepped decline in eel populations (130). Parasitized eels showed stronger levels of plasmatic cortisol and higher gill Na+/K+—ATPase activity that added up to physical constraints (salinity, temperature) to mark female eels in the last stage of silvering to be more prone to be stressed by the combined effects of several stressors. The synergistic effects of parasitism, hypoxia and biotic factors included in the analysis of eel physiology signal the way by which a comprehensive and realistic study of stress responses should be performed. In anadromous salmonids, for instance, long-lasting migrations subdue the cortisol resistance and chronically stress semelparous species. To date, the crosstalk between immune and hormonal components remains unsolvable due to the complexity of the activation/suppression interplay between cortisol, thyroid, growth and sex hormones, B cell lymphopoiesis, inflammation, antibody responses and the development of immunological memory at different stages of their life cycle (131). In this case, the stressotope demands a pronounced level of multiscale complexity to integrate the adaptive vs. maladaptive effects of stress in such migratory species.

As discussed above, fish stressotopes harbor several opportunistic and obligate parasitic, fungal, viral, and bacterial pathogens that may transmit stress-prone phenotypes vertically, by parasite colonization of gonadal tissues, and direct cortisol effects into eggs (119, 132) and affect not only broodstock and natural populations but both sexes differentially as well. Therefore, the puzzling diversity of teleost reproductive strategies may be also partially explained assuming compensatory genetic changes that overcome maladaptive responses to distressful environments. This leads to plastic reproductive adaptations between sexes to predatory and pathogenic pressures by virtue of sex-specific differences in the reproductive hormonal axis.

Overall, these and other studies imply that to accurately define a stressotope, the range of abnormal values in distress physiological adjustments, the scope of stressome components to be included in the analysis of allostatic load and the intersex differential responses to severe stressors, should necessarily be taken into account. Considering that in teleosts, as in the rest of vertebrates, steroids regulate reproductive outcomes but also metabolism, stress responses, behavior and immune function, usually in a seasonal way (133, 134), the differential effect of estrogens- and testosterone-derived mediators must be included in the stressome catalog.

## Conclusion

We have outlined some of the key processes and influences required to properly define a stressotope, ranging from the molecular to the ecological ones. Stress is a foreground concept defined against a background of interactions between network genome expression and phenome consolidation in a particular ecological niche. A stressotope approach that could help to elucidate common responses to diverse stressful scenarios is not only informative but also necessary to reduce the diversity of fish lifestyles to a minimum common set of telltales and indicators of allostatic loads originating from multiple and recurrent stressors. There is a growing shift in the literature of stress responses in fish toward a more integrate view of allostatic description. However, this approach is still hampered by the lack of analytical tools, peculiarities of fish genomes and the fuzzy definition of common inter-specific endpoints of distress-related physiological changes across behavioral phenotypes. Moreover, fish are considered more labile and diverse in their physiology than other vertebrates. We can describe teleosts as animals that indulge in sex changes, inhabit environments hostile to ectothermic metabolisms, grow indefinitely, modify their coping styles, or individualities in response to environmental and parasitic insults (135, 136), have higher rates of cell proliferation in the adult brain compared to mammals, and that are strongly dependent on the social interactions and physical environments (137, 138). Therefore, a roadmap for minimum common descriptors of stress responses, a stressotope, must be drawn considering the behavioral plasticity of teleosts, an integrative concept that harbors the cross-linked effects of neuroimmunoendocrine cross-talks that integrate in a variable set of phenotypes from specific activation of pan-specific stressomes.

## Author Contributions

JB and LT conceived and wrote the review and JB crafted the figures. Both authors contributed to manuscript revision, read, and approved the submitted version.

### Conflict of Interest Statement

The authors declare that the research was conducted in the absence of any commercial or financial relationships that could be construed as a potential conflict of interest.
